# Waardenburg–Shah syndrome: A case of neonatal palliative care

**DOI:** 10.1002/pdi3.2506

**Published:** 2024-09-24

**Authors:** Michael Colpani, Maria del Carmen Rodriguez Perez, Bruno Angelo Drera, Francesco Maria Risso

**Affiliations:** ^1^ Neonatology and Neonatal Intensive Care Unit Children's Hospital–ASST Spedali Civili Brescia Italy; ^2^ Neonatal Pathology Cremona Hospital ‐ ASST Cremona Cremona Italy

## 1 CASE PRESENTATION

**FIGURE 1:**
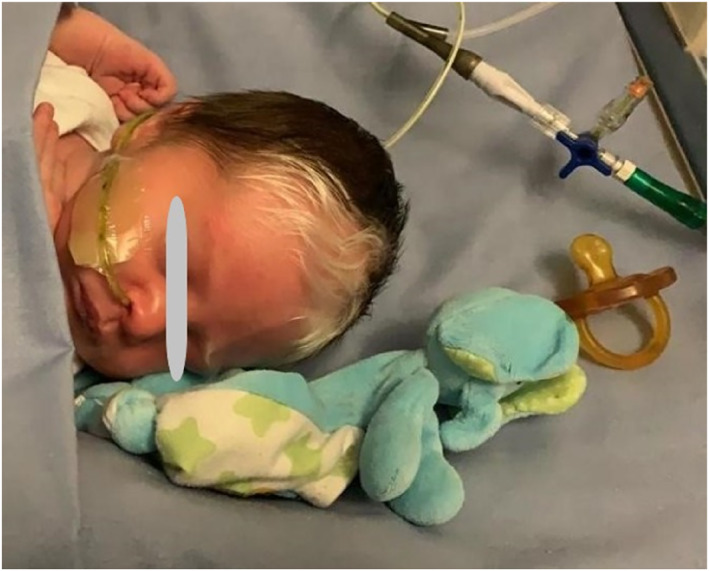
Pigmentation anomalies of the hair and eyebrows (piebaldism).

We present the case of a child born from eutocic birth induced at 37.3 weeks of gestation after a normal pregnancy up to 36 weeks when dilation and hyperechogenicity of the intestinal loops on fetal ultrasound was found. At birth, the newborn was hyporeactive and hypotonic with poor spontaneous respiratory drive for which ventilation with T‐piece device was started with recovery of tone, color and reactivity. For the appearance of projectile vomiting, aspiration of fecal secretions from the stomach was performed. The baby was transferred to the neonatal subintensive care unit, where chest‐abdomen radiography and abdomen ultrasound were performed and showed intestinal obstruction. On physical examination, pigmentation anomalies of the hair and eyebrows were found (Figure 1).

The surgical evaluation and the repeated radiological exams confirmed the bowel obstruction, therefore the nasogastric probe in aspiration was positioned. An ultrasound‐guided centrally inserted central catheter (CICC) ‐ power injectable polyurethane catheter, 3 Fr single lumen ‐ was inserted in the right brachiocephalic vein, tunneled to the ipsilateral infraclavear area and stabilized with a subcutaneous anchor system.

**FIGURE 2:**
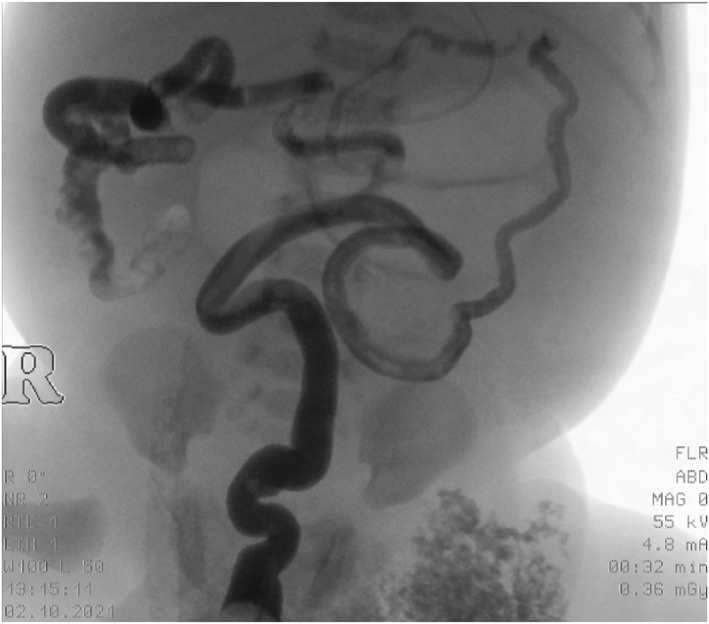
Barium enema, microcolon with considerable dilatation of the intestinal loops.

On the first day of life, exploratory laparotomy was performed. The barium enema performed pre‐surgery showed the presence of microcolon with considerable dilation of the intestinal loops (Figure 2).

During the operation, at the level of the jejuno‐ileal passage, there was a reduction in the diameter of the loops with the presence of clear meconium of increased and sticky consistency downstream and biliary intestinal content upstream. Washing the loops with 10% acetylcysteine solution allowed the recanalization of the hive with complete elimination of meconium.

In the first postoperative day, considering the good gas exchange and clinical stability, the baby was extubated and placed in non‐invasive ventilation. On the second postoperative day, abundant biliary secretions persisted in the gastric area. Radiographs showed persistence of contrast medium administered preoperatively. The Transient Evoked OtoAcoustic Emissions and automated auditory brainstem response otoemissions were bilaterally absent, while the ophthalmological examination did not reveal chorioretinal anomalies.

Suction rectal biopsies were performed on suspicion of Hirschsprung‐associated disease. The absence of mature ganglion cells of the submucosal nerve plexus with widespread nervous hypertrophy confirmed the diagnosis of aganglionosis.

Because of the marked abdominal distension, post‐lavage white meconium leakage, and abundant gastric biliary secretions from the tube, a second exploratory laparotomy was performed. During the operation, multiple biopsies were performed in each intestinal segment from the ileocecal valve to the stomach which showed complete aganglia from the colon to Treitz on extemporaneous histological examination.

The clinical picture characterized by sensorineural deafness, pigmentation abnormalities and complete aganglionosis pointed towards Waardenburg type 4 syndrome. Genetic exam (next generation sequencing) was therefore performed in the child and the parents which revealed the presence of the homozygous mutation in the *EDN3* gene (p.Arg96Cys; c.286 C > T), configuring the subtype 4B.

Taking into account the clinical picture, the prognosis and the difficulty of home management, it was decided with parents to start palliative care.

The intravenous analgesic therapy (acetaminophen and fentanyl) was established on the basis of the pain scales, the discomfort assessed, and the quality of sleep. The baby was alert with good interaction for the first 4 weeks. Subsequently, palliative sedation (midazolam and morphine) was gradually introduced due to the progressive worsening of the general conditions and the intestinal picture. The baby died at 59 days of life.

## 2 DISCUSSION

Waardenburg syndrome is an auditory‐pigmentary disorder that exhibits a varying combination of sensorineural hearing loss and abnormal pigmentation of the hair, skin or eyes.^1^ Waardenburg‐Shah type 4 (WS4) is characterized by sensorineural hearing loss, heterochromia iridis, hypopigmentation, white forelock and synophrys and the presence of Hirschsprung's disease.^1^, ^2^, ^3^

Waardenburg–Shah syndrome or WS4 is genetically heterogeneous.^3^, ^4^ Type 4A is caused by a mutation in the *EDNRB* gene.^5^ 4B is caused by a mutation in the *EDN3* gene on chromosome 20q13,^5^ and 4C is caused by a mutation in the *SOX10* gene on chromosome 22q13.^6^ The p.Arg96Cys variant of the *EDN3* gene is described in the literature to be associated with Waardenburg syndrome.^4^ It is extremely rare, and appears to cause structural/functional damage to the protein. The parents are heterozygous for this particular mutation and do not present syndrome‐related signs and symptoms, while the homozygous child developed the severe form of the disease, therefore we suggest the autosomal recessive inheritance.

The therapeutic challenges represented above are all by the management of the intestinal framework. The therapy is exclusively surgical and uses the removal of the segment affected by the absence of the plexuses with the creation of an enterostomy/colostomy. The greater the extent of the removed tract is, the more severe pictures of malnutrition requiring total parenteral nutrition are likely. In our case, the intestinal segment involved extended from the rectum to the Treitz, therefore, it would have been necessary to remove the entire intestine, a condition incompatible with life.

The possibility of intestinal transplant was therefore considered, however, after appropriate evaluations and in agreement with the family, it was excluded. Bowel transplantation often requires liver transplantation to ensure digestive function.^7^ The procedure is fraught with infectious and vascular complications, and immunosuppressive therapies are also needed to avoid rejection and graft versus host disease.^7^, ^8^ Intestinal transplantations are characterized by the frequent need for subsequent transplants, in the face of a modest life expectancy after transplantation. The transplantation success rate is less than 40% at 5 years. In this case, a further problem was the patient's age, as the availability of compatible organs suitable for newborns is extremely rare and surgery is not done in such small infants. Moreover, total parenteral nutrition performed during the pre‐transplant period is burdened by the increased risk of infections, malnutrition, thrombosis and cholestasis, and by an important physical and emotional burden of the parents at home. In addition, the use of a cochlear implant would have been necessary for sensorineural deafness.

Neonatal palliative care aims at improving the quality of life when the patient's prolongation of life is no longer the goal of care or the complexity of the medical condition is associated with uncertain prognosis.^9^ A state of comfort is reached when some basic needs are met (bonding, maintenance of body temperature, relief of hunger/thirst, relief of pain/discomfort), which in this case has been facilitated by the presence of the CICC that has allowed adequate hydration and pain management. When comfort becomes the exclusive or relevant goal of treatment, multidisciplinary care is essential, given the complex needs of these infants and their families.^9^ Provision of emotional, psychological and spiritual support for families fosters a good relationship between staff and family and enables the healing of parental grief and pain. Compassionate communication and information sharing that respect parents' sociocultural beliefs and values, as well as supporting parental choice itself, have a significant impact on parental participation in the process.

## 3 CONCLUSION

Shah‐Waardenburg syndrome is a very rare syndrome with high morbidity and mortality in the neonatal age group due to total intestinal aganglia. Timely diagnosis of the disease does not ensure a favorable prognosis, as the management of the intestinal framework is complex. The presence of total aganglionosis is related to a poor prognosis, as multiorgan transplantation is associated with high infectious risk, surgical complications, metabolic alterations and poor quality of life. In these cases, palliative care can be considered in order to provide a more humane management of the newborn and to spare the patient and their family from significant physical and psychological suffering. The clinical‐therapeutic approach must therefore be tailored to guarantee a dignified quality of life.

## AUTHOR CONTRIBUTIONS

Dr Michael Colpani designed the study, collected data, drafted the initial manuscript, and reviewed and revised the manuscript. Dr Maria del Carmen Rodriguez Perez, Dr Bruno Angelo Drera and Dr Francesco Maria Risso conceptualized the study, supervised data collection, corrected initial manuscript, reviewed and revised the manuscript, coordinated and supervised data collection and critically reviewed the manuscript for important intellectual content. All authors approved the final manuscript as submitted and agreed to be accountable for all aspects of the work.

## CONFLICT OF INTEREST STATEMENT

The authors have no conflicts of interest to disclose.

## ETHICS STATEMENT

Hospital review board exempts case report from ethics committee approval.

## Data Availability

Data sharing is not applicable to this article as no new data were created or analyzed in this study.
